# The pathogenic roles of lncRNA-Taurine upregulated 1 (TUG1) in colorectal cancer

**DOI:** 10.1186/s12935-022-02745-1

**Published:** 2022-11-04

**Authors:** Shirin Azizidoost, Ava Nasrolahi, Farhoodeh Ghaedrahmati, Bartosz Kempisty, Paul Mozdziak, Klaudia Radoszkiewicz, Maryam Farzaneh

**Affiliations:** 1 Atherosclerosis Research Center, Ahvaz Jundishapur University of Medical Sciences, Ahvaz, Iran; 2 Infectious Ophthalmologic Research Center, Ahvaz Jundishapur University of Medical Sciences, Ahvaz, Iran; 3 Department of Immunology, School of Medicine, Isfahan University of Medical Sciences, Isfahan, Iran; 4 Graduate Physiology Program, North Carolina State University, 27695 Raleigh, NC USA; 5 Translational Platform for Regenerative Medicine, Mossakowski Medical Research Institute, Polish Academy of Sciences, 02-106 Warsaw, Poland; 6 Fertility, Infertility and Perinatology Research Center, Ahvaz Jundishapur University of Medical Sciences, Ahvaz, Iran

**Keywords:** Colorectal cancer, LncRNAs, TUG1, Tumorigenesis

## Abstract

Colorectal cancer (CRC) is a gastrointestinal tumor that develops from the colon, rectum, or appendix. The prognosis of CRC patients especially those with metastatic lesions remains unsatisfactory. Although various conventional methods have been used for the treatment of patients with CRC, the early detection and identification of molecular mechanisms associated with CRC is necessary. The scientific literature reports that altered expression of long non-coding RNAs (lncRNAs) contributed to the pathogenesis of CRC cells. LncRNA TUG1 was reported to target various miRNAs and signaling pathways to mediate CRC cell proliferation, migration, and metastasis. Therefore, TUG1 might be a potent predictive/prognostic biomarker for diagnosis of CRC.

## Introduction

Colorectal cancer (CRC) is a gastrointestinal malignancy, ranked as the third most commonly diagnosed, and the second cause of cancer mortality worldwide [1]. This type of cancer originates from the colon, rectum, or appendix [2]. Pieces of evidence showed that the CRC incidence rate has risen during the past decades, specifically in developing countries [3]. Over 1.85 million new CRC cases are reported annually, with an increasing number of young people before the age of 50 [4]. Several factors such as genetics, epigenetics, and environment distributed across the CRC etiology and are responsible for disease heterogeneity [5, 6]. Etiologically, the three patterns of disease onset are sporadic, familial, and hereditary forms that affected 70%, 25%, and 5% of patients [7, 8]. It has been found that the age, environmental factors, dietary, lifestyle, gut microbiota, and genetic changes predispose persons to CRC [9]. Current treatment of CRC in primary and metastatic patients include laparoscopic surgery for primary disease, resection in case of metastatic tumors, radiotherapy for rectal neoplasm along with palliative, and neoadjuvant chemotherapies [10]. Besides, antibodies, probiotics, agarose tumor macrobeads, and gold-based drugs or their combinations are used for patients with CRC [11]. It has been found that the combination of chemotherapy and anti-EGFR (epidermal growth factor receptor) monoclonal antibodies cetuximab and panitumumab can prolong the median survival rate of these patients by 2 to 4 months compared with chemotherapy alone [12]. However, the impact of these therapies on the 5-year survival remains limited and very expensive [13, 14]. Several mutations in oncogenes, tumor suppressor genes, and genes associated with DNA repair have been identified as genetic risk factors for CRCs [15]. It has been reported that genetic mutations in SMAD4, BRAF, KRAS, PIK3CA, SMAD2, PTEN, and c-MYC play essential roles in patients with CRC [11]. Recent studies demonstrated that long non-coding RNAs (lncRNAs) as a subgroup of RNAs longer than 200 nucleotides presented important functions in the pathogenesis of CRC [16–18]. Aberrant expression of lncRNA is associated with several diseases, as well [17]. Previous investigations reported that lncRNA TUG1 showed tumor-suppressive or oncogenic functions in different types of cancers [19, 20]. Studies revealed that TUG1 expression was increased in CRC tumor tissues and promoted cell proliferation [21–23]. Further analysis revealed a significant negative correlation between the levels of TUG1 and the overall survival rate of patients with CRC [24]. In this review, we summarized functional roles of this lncRNA in the tumorigenesis of CRC cells.

### Biological properties of lncRNA TUG1

**Fig. 1:**
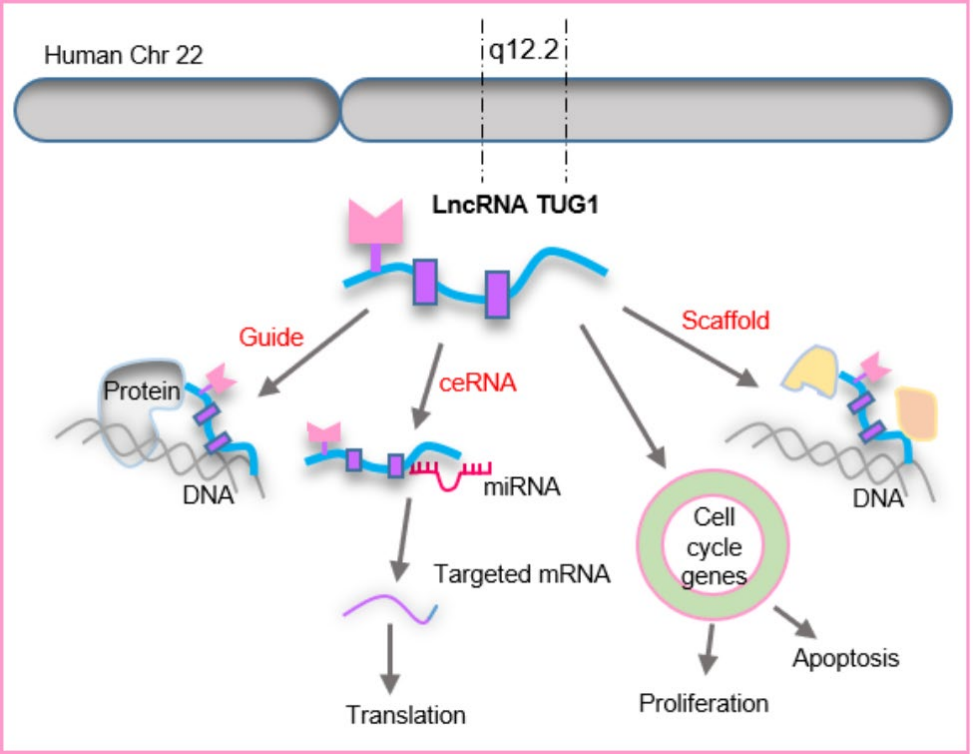
Multiple functions of lncRNA TUG1. Taurine-upregulated gene 1 (TUG1) is a 7.1-kb lncRNA that in the human genome is located on chromosome 22q12.2. TUG1 can function as a scaffold, cell-cycle regulator, guide, and ceRNA (competing endogenous RNA).

An initial genomic screening for genes upregulated in response to taurine treatment in developing mouse retinal cells detected taurine-upregulated gene 1 (TUG1) (also known as TI-227 H, LINC00080, and NCRNA00080), a 7.1-kb lncRNA that in the human genome is located on chromosome 22q12.2 [25]. Functional studies revealed mice lacking TUG1 had impaired retinal development [26]. There is also evidence that TUG1 is transcriptionally regulated by p53 [27]. The polycomb-repressive complex 2 (PRC2) contains enhancer of zeste homologue 2 (EZH2), suppressor of zeste 12 (SUZ12), and embryonic ectoderm development (EED) [28] that catalyzes lysine residue 27 di- and trimethylation on histone 3 (H3K27me3) to repress gene expression [29]. TUG1 by recruiting and binding to the PRC2 complex functions as a dynamic scaffold [30, 31]. TUG1 knockdown induced an upregulation of the cell-cycle genes, suggesting that TUG1 is involved in both cell proliferation and apoptosis [22]. TUG1 as a potent epigenetic regulator can mediate histone modification and DNA methylation in target genes [24, 32]. Besides, TUG1 by acting as a competing endogenous RNA (ceRNA) could sponge and repress microRNA (miRNA) [33]. Figure 1 displays different functions of TUG1.

TUG1 has recently been proposed as an oncogene in several types of cancer [34–36]., TUG1 is associated with large tumor size, advanced pathological stages, and distant metastasis [37, 38]. Experimental studies have disclosed that TUG1 significantly stimulated tumor cell proliferation, invasion, colony formation, and drug resistance in CRC cells (Table 1). Overexpression of TUG1 via mediating epithelial-mesenchymal transition (EMT)-associated gene expression, reduction of E-cadherin expression, and boosted the vimentin, N cadherin, and fibronectin expression promoted the invasion and metastasis of CRC cells [24, 39, 40].

**Table 1:** Pathogenic roles of lncRNA TUG1 in colorectal cancer

Axis	Cell line (in vitro)	Animal model (in vivo)	Patients-derived tissue	Cancer Chemo-resistance	Cancer initiation (cell proliferation)	Cancer progression	Cancer metastasis	Cancer apoptosis	Refs.
miR-145-5p/TRPC6	✓	✓	✓	---	✓	✓	---	---	[42]
miR-138-5p/ZEB2	✓	---	✓	---	---	✓	✓	---	[43]
Wnt/β-catenin	✓	✓	✓	---	✓	---	---	---	[46]
GATA6/BMP	✓	✓	---	✓	---	---	---	✓	[67]
miR-153-1/KLF4	✓	✓	✓	----	✓	---	✓	---	[60]
miR-186/CPEB2	✓	---	✓	✓	---	---	---	✓	[69]
TGF-β/TWIST1	✓	✓	✓	---	---	---	✓	---	[49]
miR-197-3p/TYMS	✓	✓	✓	✓	✓	---	---	✓	[65]
miR-421/KDM2A/ERK/SP1	✓	✓	✓	---	---	✓	---	✓	[51]
miR-542-3p/TRIB2/Wnt/β-catenin	✓	✓	✓	---	✓	✓	---	✓	[58]
miR-195-5p/IGF2BP2	✓	✓	✓	✓	---	---	---	---	[73]

### The critical TUG1/miRNAs/transcription factors axes in colorectal cancer

**Fig. 2:**
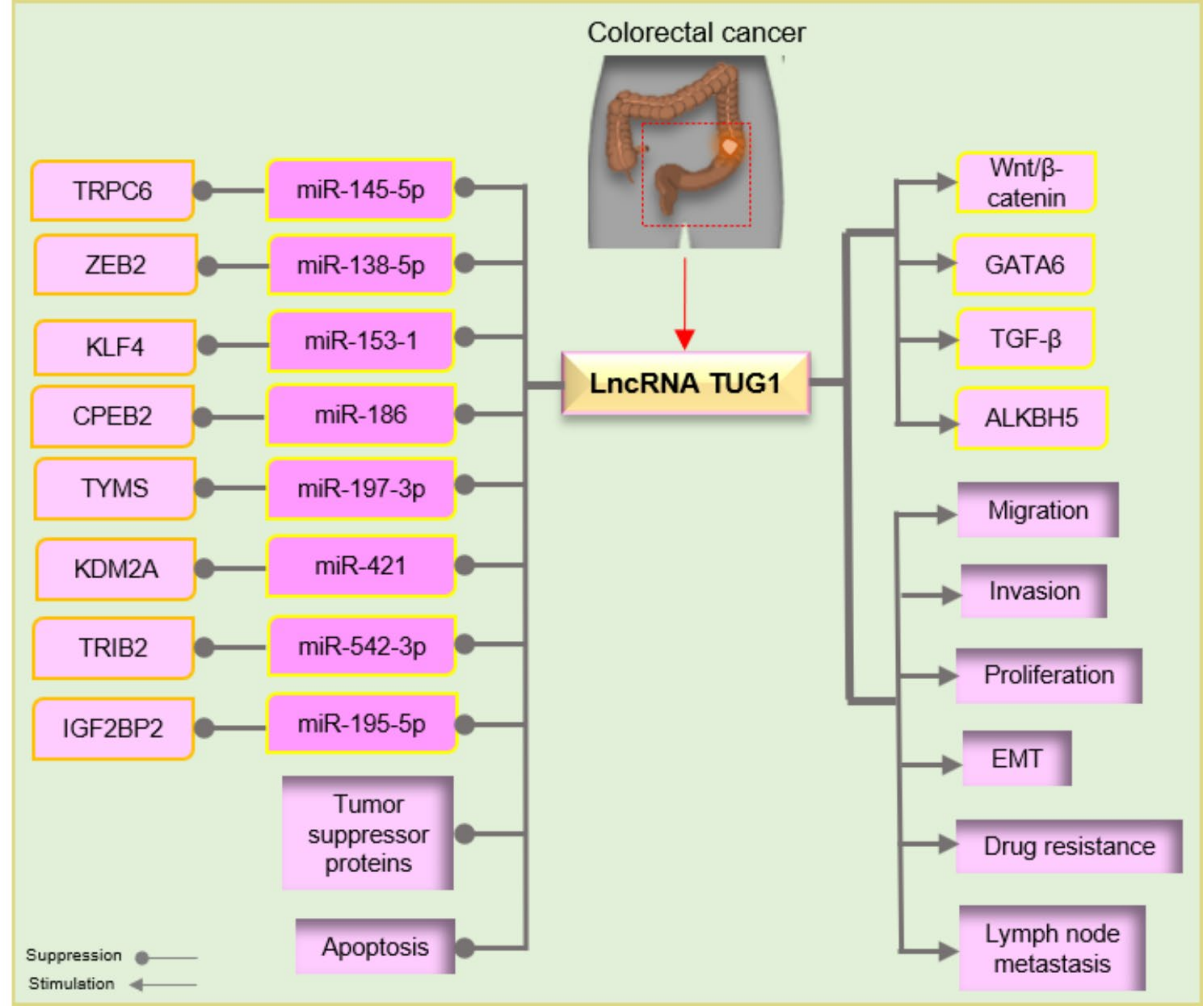
LncRNA TUG1 by targeting various miRNA/mRNA axes presented essential role in colorectal cell tumorigenesis

There is growing evidence that TUG1 by targeting several signaling pathways plays critical roles in the progression of CRC cells [24]. Here, we described multiple miRNAs/transcription factors axes (Fig. 2) that can be regulated via this lncRNA in CRC.

#### TUG1/miR-145-5p/TRPC6

High expression of TUG1 has been proved to be correlated with CRC pathogenesis including proliferative along with the migratory ability, cell viability, tumor growth, and subcutaneous tumor formation [41, 42]. TUG1 is known to interact with miR-145-5p and regulate CRC cellular processes. Moreover, miR-145-5p suppressed the expression transient receptor potential cation channel subfamily c member 6 (TRPC6) as its candidate target, which its overexpression brought back miR-145-5p function in CRC. Altogether, TUG1 induces progression of CRC through miR-145-5p/TRPC6 axis, thereby regarding as a possible diagnostic marker for CRC management [42].

#### TUG1/miR-138-5p/ZEB2

It has been shown that high expression of TUG1 is correlated with increased proliferation and invasion along with reduced apoptosis of CRC cells [43]. TUG1 overexpression was also closely associated with the overall survival of CRC patients, indicating TUG1 as poor prognosis biomarker for CRC [44]. Zinc finger E-box binding homeobox 2 (ZEB2) is a binding protein that participated in CRC metastasis and associated with human CRC poor prognosis [45]. There was a positive and negative correlation between TUG1 with ZEB2 and miR-138-5p, respectively. Low expression of ZEB2 or overexpression of miR-138-5p reversed the induction of EMT which was caused by TUG1 overexpression. Therefore, TUG1 by suppressing the miR-138-5p/ZEB2 axis facilitated CRC occurrence and metastasis [3, 41, 43, 44].

#### TUG1/Wnt/β-catenin

High expression of TUG1 has been implicated in clinicopathological features of CRC including advanced tumor stage along with reduced overall survival and disease-free survival [24, 44, 46]. The Wnt/β-catenin signaling was found to transcriptionally regulate CRC proliferation [47]. TUG1 silencing resulted in low activity of Wnt/β-catenin and suppression of proliferation. In the CRC xenograft model, low expression of TUG1 inhibited both tumorigenicity and β-catenin nuclear localization. TUG1 through changing the nuclear localization of β-catenin reduced the Wnt/β-catenin signaling activity and subsequent induced CRC proliferation. Therefore, TUG1/Wnt/β-catenin could exert promising advancement for CRC prevention [44, 46].

#### TUG1/TGF-β/TWIST1

Transforming growth factor-beta (TGF-β) is involved in CRC tumorigenesis [48]. A recent study reported that TGF-β induced migration of CRC and overexpressed TUG1 as its downstream molecule. Recent findings demonstrated that TUG1 blockade suppressed migration, invasion, in vitro EMT along with in vivo lung metastasis. Hence, TUG1 is necessary for TGF-β-promoted pathophysiological features of CRC [49]. Twist family BHLH transcription factor 1 (TWIST1) stands as a kind of transcriptional modulator which is activated by TGF-β, resulting in low expression of E-cadherin [50]. TWIST1 silencing using siRNA resulted in significant reduction of CRC migration and EMT. It can be concluded that TGF-β-induced metastasis of CRC is regulated through the TUG1**/**TWIST1/EMT network, highlighting TUG1 as a novel target to inactivate the TGF-β signaling [49].

#### TUG1/miR-421/KDM2A/ERK/SP1

Specificity protein 1 (SP1) has a positive-regulated manner with TUG1 in CRC cells [51]. SP1 as an oncogene was found to promote CRC progression and metastasis [51, 52]. TUG1 loss of function inhibited cell growth and induced apoptosis of CRC cells [51]. Growing evidence revealed a negative correlation between TUG1 and miR-421 as a CRC tumor suppressor factor [53, 54]. Lysine demethylase 2 A (KDM2A) is a CRC oncogenic gene and a target for miR-421. TUG1 by sponging miR-421 induced KDM2A expression [51, 55]. Moreover, TUG1 has been found to intensify in vitro progression of CRC through the ERK signaling. Also, SP1 promoted in vivo CRC tumorigenesis by miR-421 suppression and KDM2A induction through TUG1 overexpression. Altogether, TUG1 as an oncogene can interact with SP1 and the miR-421/ KDM2A/ERK axis to facilitate CRC progression [51].

#### TUG1/miR-542-3p/TRIB2/Wnt/β-catenin

Tribbles homolog 2 (TRIB2) is an atypical protein kinase that has been dramatically upregulated with TUG1 in CRC tissues and cells [56, 57]. High expression of TUG1 by suppressing miR-542-3p was associated with tumor stage, lymph node metastasis, and histological differentiation of CRC patients. TUG1 or TRIB2 loss of function prohibited proliferation, migration, invasion along with in vivo tumor growth but facilitated CRC apoptosis. Besides, upregulation of TRIB2 as a miR-542-3p target reversed the impact of TUG1 silencing on CRC progression [58]. Considering the role of the Wnt/β-catenin signaling in CRC development, miR-542-3p suppression or TRIB2 upregulation has been reported to partly bring back the inhibitory function of TUG1 knockdown on the Wnt/β-catenin signaling. ThereforeTUG1 is regarded as a tumor promoter that stimulated CRC pathogenesis and drug resistance through the miR-542-3p/TRIB2 axis [58, 59].

#### TUG1/miR-153-1/KLF4

In contrast to highly-expressed TUG1 in CRC, miR-153-1 was under expressed. Depletion of TUG1 using si-TUG1 as well as ectopic expression of miR-153-1 repressed the proliferative and migratory capacity of CRC cells. Also, upregulated TUG1 reversed miR-153-1-mediated suppression of CRC cells [60]. Kruppel-like factor 4 (KLF4) is a zinc finger transcription factor that plays as a tumor suppressive gene in CRC [61]. Recent findings identified KLF4 as a direct transcription factor for miR-153-1 can suppress CRC pathogenesis but its expression negatively modulated by TUG1. Interestingly, TUG1-deficient mice demonstrated high and low expressions of E-cadherin along with N-cadherin as tumor metastasis-correlated EMT markers exerting the TUG1/miR-153-1/KLF4 axis in in vivo EMT of CRC cells. Such regulatory axis might provide great insights for either diagnostic or treatment possibility of CRC [60].

### Chemoresistance features of TUG1

Chemotherapy in combination with targeted therapy has been found to impair tumor recurrence and increase survival rate of CRC patients, but chemotherapeutic resistance is considered as the leading cause of CRC therapy failure.Therefore, molecular knowledge of chemotherapeutic resistance is required for CRC tumor biology [62, 63]. 5-fluorouracil (5-FU) is regarded as an effective first-line drug for CRC patients, but unknown molecular approaches are still complicated its recovery features [17, 64]. Accumulating data demonstrated that TUG1 is overexpressed in 5-FU resistant CRC tissue and cells, which were associated with poor prognosis. TUG1 blockade was implicated to dramatically promote CRC cells sensitive to 5-FU through suppressing CRC cell apoptosis which is regulated by miR-197-3p and TYMS as a direct target of miR-197-3p. Such findings highlighted the possible significance of TUG1 as a predictive marker for exerting CRC response to 5-FU therapy and indicated TUG1 silencing as a novel therapeutic approach to reverse 5-FU resistance [65]. Besides, cancer stem cells are shown to be involved in CRC chemoresistance [66]. It has been found that TUG1 silencing inhibited CRC stem cell resistant to oxaliplatin through reducing GATA6 and targeting the BMP pathway. Altogether, TUG1 promoted CRC stem cell features and chemotherapeutic resistance via inducing the stability of the GATA6 protein, providing promising insights for CRC clinical therapy [67]. Improvement of drug resistant sensitivity remains an immediate necessity for CRC chemotherapies. CRC resistance to methotrexate (MTX) as the earliest cytotoxic drugs is still a main challenge to the physicians [62, 68]. A recent study indicated that TUG1 repressed CRC cell sensitivity to MTX through targeting its interaction with miR-186. TUG1 blockade re-sensitized CRC cell resistant to MTX. Indeed, a negative correlation between miR-186 and the cytoplasmic polyadenylation element binding protein 2 (CPEB2) protein has been shown in MTX resistant tumors. Therefore, TUG1 regulated CRC resistant to MTX through targeting miR-186 and consequent induction of CPEB2 expression, thereby holding TUG1 as a possible target for CRC management [69].

Insulin-like growth factor-2 mRNA–binding protein (IGF2BP) family members as a kind of RNA-binding proteins participated in tumorigenesis as well as chemoresistance via influencing either stability, translatability, or localization of lncRNA [70–72].IIt has been found that TUG1 and IGF2BP2 were both high-expressed in CRC cell resistant to cisplatin through autophagy activation. Low TUG1 expression decreased CRC chemoresistance to cisplatin and facilitated miR-195-5p expression. Therefore, the TUG1/IGF2BP2/miR-195-5p axis intensify CRC cell growth and induce such malignant cell resistance to cisplatin, regarding as underlying target for CRC therapy [73].

### Prognostic significance of TUG1 in colorectal cancer

Along with the biological behaviors of TUG1 in regulating CRC pathogenesis, it is also emerging as a crucial substrate for the progress of CRC biomarkers for early detection, prognosis prediction, and anticipating therapy response to diverse chemotherapies and developing therapies [74]. Currently, a study proposed that TUG1 could play a vital function in CRC metastasis. following investigation of the TUG1 expression levels in 120 CRC patients, high TUG1 expression was observed in tumor tissue which was closely associated with the poor survival time of the CRC patients [24, 75, 76]. Further in vitro experiments revealed the oncogenic impact of TUG1 upregulation in CRC cell lines. In the xenograft animal model, increased expression of TUG1 stimulated colony formation, migratory ability, and metastatic potential. Indeed, the researchers observed that TUG1 activated EMT-correlated gene expression [24, 77]. Another study proposed that the highly-expression of TUG1 was a CRC convinced unfavorable prognosis marker [78]. Cumulatively, TUG1 may act as a prognostic biomarker and a curative target. With more attempts affirm to the study of lncRNA particularly TUG1, it is optimistic that TUG1 will finally attain clinical utility [79]. In contrast, a recent study on 47 CRC patients indicated that there were no remarkable correlations between TUG1 expression and clinicopathological features of CRC. Besides, TUG1 expression could not forecast the overall survival and progression-free survival in CRC patients [80].

## Conclusion

LncNAs can be used as biomarkers for the diagnosis, prognosis, and monitoring of the progression of the disease because of their tissue-specific expression and high stability [81]. Several studies reported that lncRNAs are closely linked to a variety of cancer types and might function as oncogenes or oncogene suppressors depending on the type of cancer [82, 83]. Although the role of TUG1 in the characteristics and chemoresistance of CRC stem cells is still not well-defined, it has been already presented as an attractive potential biomarker because of its tumor-promotive function via diverse mechanisms, such as RNA-RNA and RNA/transcription factors interactions [46, 84]. It was investigated by the same group that TUG1 increased the characteristics and oxaliplatin resistance of CRC stem cells by enhancing GATA6 stability [14]. TUG1 is suggested to solve the problem of fluoropyrimidine (Fu)-based chemotherapy. TUG1 appears to mediate 5-Fu resistance in CRC through the miR-197-3p/TYMS axis [9]. Knockdown of TUG1 resensitized resistant cells to the exposure of 5-Fu and induced cell apoptosis. This lncRNA by targeting miR-186 stimulated CPEB2 to mediate methotrexate resistance in CRC [15]. Taken together, the role of lncRNA TUG1 in CRC drug resistance seems to be crucial and holds great promise as a potential therapeutic target. Current findings regarding TUG1 not only promote a better understanding of CRC pathogenesis and development but also facilitated the progress of cancer lncRNA therapy. However, many mechanisms remain poorly described suggesting a great need for further study.

## Data Availability

The datasets used and/or analyzed during the current study are available from the corresponding author on reasonable request.
